# A Real-Time Monitoring Method for Civil Aircraft Take-Off and Landing Based on Synthetic Aperture Microwave Radiation Technology

**DOI:** 10.3390/s22103675

**Published:** 2022-05-12

**Authors:** Houcai Chen, Junxiang Ge, Deqing Kong, Zhenwei Zhao, Qinglin Zhu

**Affiliations:** 1 Department of Electronics and Communication, Nanjing University of Information Science and Technology, Nanjing 210044, China; jxge@nuist.edu.cn; 2 China Research Institute of Radiowave Propagation, Qingdao 266107, China; zhaozw@crirp.ac.cn (Z.Z.); zhuql1@crirp.ac.cn (Q.Z.); 3 National Astronomical Observatories, Chinese Academy of Sciences, Beijing 100045, China; kdq@bao.ac.cn

**Keywords:** synthetic aperture, radiometer, inverse imaging, error correction, civil aircraft, auxiliary landing

## Abstract

It is important to monitor the take-off and landing of civil aircraft using passive detection methods. Due to the strict aircraft safety requirements and the electromagnetic environment around an airport, using too many active detection methods should be avoided. Using an aircraft’s microwave radiation signal detection is very advantageous because it does not actively emit signals and has a strong cloud penetration, suitable for all-weather observation. This paper introduces a synthetic aperture microwave radiation system for monitoring the take-off and landing of civil aircraft, which is characterized by real-time two-dimensional imaging, and the image refresh rate can reach 10 ms, which meets the high refresh rate requirements for aircraft imaging. Applicable system parameters and antenna array distribution scheme and imaging algorithm are given. Then the paper focuses on the error analysis and correction method of the system. The correction method is simple and fast, which avoids the disadvantage that the error needs to be corrected regularly in the laboratory environment, and is suitable for airport application. Finally, the simulation and experimental results show that this technology can be used for real-time monitoring of civil aircraft during take-off and landing, and it is a practical means to assisting landing.

## 1. Introduction

Due to the advantages of all-weather imaging that does not employ mechanical scanning, synthetic aperture microwave radiation measurement technology is attracting more and more attention. Comprehensive aperture microwave radiation measurement technology originated from radio astronomy research, and its basic idea is to replace direct measurements in the space domain using a traditional radiometer with measurements of the space-frequency domain, which has the advantages of better resolution. Since ESTAR (the synthetic aperture radiometer) was developed in the late 1980s [1], extensive research has been conducted in the United States and Europe. MIRAS (Microwave Imaging Radiometer with Aperture Synthesis) [2,3,4], GeoSTAR (Geostationary Synthetic Thinned Array Radiometer) [5,6], and other high-performance interferometric microwave radiometers have been successfully developed. ESTAR airborne tests have been used to acquire a large number of experimental data and for the inversion of soil moisture, seawater salinity, and other parameters, and MIRAS onboard SMOS has been used to observe the soil moisture with a spatial resolution of more than 50 km and seawater salinity with a spatial resolution of more than 200 km worldwide. GeoSTAR was the first sounding instrument used in geosynchronous orbit for microwave brightness and moisture detection and rainfall observation. Overall, the development of synthetic aperture radiometer is still mainly airborne and spaceborne, mainly used for earth observation of soil moisture and seawater salinity, with a resolution of about 50 km. In terms of ground detection, C. Zhang et al. has done a lot of research on the imaging theory of RS-SAIR, and successfully developed the SARS—5 imager. The imaging results of the imager have excellent performance [7]. Huazhong University of Science and Technology introduced a one-dimensional prototype of the millimeter-band Aperture Synthesis Radiometer (ASR) [8,9,10]. ASR can generate one-dimensional brightness temperature images of natural scenes. To generate two-dimensional images, a rotating stage is used to provide scans in another dimension. ASR produces good images of natural scenes such as the sun and buildings. The system works in the 8 mm frequency band, the bandwidth is 10 MHz, and the integration time is 32 ms. The system takes several seconds for image reconstruction of stationary objects (including mechanical scan time). In terms of aviation exploration applications, NASA has developed a high-performance optical mechanical scanning millimeter wave imager working at 94 GHz, which can carry out high-resolution imaging (super-resolution), and can be used to monitor the ground motion of aircraft in bad weather conditions [11,12]. It uses two counter rotating mirrors to simulate the linear scanning of a single high-speed large aperture flapping mirror. The observed temperature sensitivity of the imager is about 2 K, and its spatial resolution is slightly lower than the theoretical prediction. It is characterized by designing and manufacturing a reflective lens with light weight and low loss, which is combined with rotary scanning to form a wide-angle reflective scanning antenna, so as to complete two-dimensional scanning imaging.

Although previous scholars have done a lot of research on synthetic aperture technology in spaceborne and ground-based applications, it is rare to use this technology for real-time detection of flying targets from the airport. In this connection, the trade-off values such as detection distance, spatial resolution, and integration time need to be considered. Our research gives a reasonable key index design and a special circular array, and deduces a simple and convenient error compensation method. The original airborne and spaceborne technologies were extended to ground applications, and a quasi-real-time 2D measurement system was designed for detecting flying targets (without any mechanical scanning). System sensitivity, measurement time, and hardware cost are balanced. The main goal is to design a fully passive detection system suitable for use in airports by using synthetic aperture microwave radiometer technology to realize real-time imaging monitoring of aircraft in special weather conditions such as cloudy and foggy days. For civil aircraft detection applications, synthetic aperture radiometer technology can be used in a sparse small-aperture antenna array to achieve observations similar to those of large physical observation aperture antenna, which can reduce the size and weight of the antenna and enable real-time imaging without mechanical scanning. At the same time, the use of microwave band measurements can reduce the influence of clouds and fogs on the detection target. These advantages make the system more suitable for practical applications and improve its application prospects.

Unlike the direct power measurement and imaging principle of traditional radiometers, synthetic aperture radiometric imaging samples the radiance-temperature distribution in (angular) frequency domain with the aid of interferometric data to obtain discrete values of the so-called visibility function. Then, this function is inverted to retrieved a brightness temperature map of the scene observed by the antenna array. Aperture synthesis reduces the signal-to-noise ratio of each measurement due to the use of smaller antenna apertures. However, since no mechanical scanning is required, the sensitivity of a single measurement can be increased by increasing the integration time. According to discussions in other literatures, even with minimal redundant arrays [1,13,14], it is possible to obtain radiation sensitivity close to the equivalent real aperture radiometer.

When designing the system, in addition to obtaining the ideal system resolution as much as possible, it is very important to select the appropriate atmospheric window. The transmittance of microwave window below 20 GHz is close to 100%, so it is feasible for us to choose 15.2 GHz as the working frequency band of airport application. The conventional working frequency band is not selected, because the active detection equipment has used higher resolution frequency bands such as 94 GHz, and other frequency bands such as 20~30 GHz are greatly affected by atmospheric water vapor and liquid water. It should also be emphasized that using techniques based on radiative transfer principles will inevitably suffer from rain attenuation, including the 15.2 GHz chosen in this design. In the case of heavy rain, the brightness temperature of rainwater received by the system may exceeds 200 K. The aircraft signal becomes smaller than the rainfall signal after attenuation, so the brightness temperature of the aircraft cannot be effectively measured. Therefore, synthetic aperture microwave radiometry is not suitable for rainy days. In order to obtain better measurement results when there are only a few antennas, synthetic aperture radiometers for remote sensing and other applications usually employ non redundant antenna arrays. When the number of antennas is determined, the non-redundant array can maximize the number of spatial frequency sampling points, so as to improve the antenna inversion effect. After determining the arrangement of the antenna array, calculating the brightness temperature of the measured object with an appropriate inverse imaging algorithm and error correction technology is the key to developing a passive detection system using synthetic aperture radiometer technology.

In this connection, this paper summarizes the research results of synthetic aperture microwave radiation measurement technology in spaceborne and ground detection, and discusses the frequency selection and so on. In the following paper, we give a system design scheme, focusing on the antenna array arrangement and the correction method of system error when detecting aircraft on the ground.

## 2. Materials and Methods

### 2.1. System Design

Microwave radiometer is a passive remote sensing device which can measure the microwave radiation energy of objects. Under the condition of actual aperture, the improvement of imaging resolution can only be achieved by increasing the physical aperture of the antenna. In order to realize field of view observation, a mechanical scanning device is usually required. For fast moving targets such as aircraft, using mechanical scanning for fast tracking and measurement is neither economical nor effective. Therefore, the synthetic aperture microwave radiometry technology is proposed to realize the quasi real-time passive imaging of aircraft. One of the main advantages of synthetic aperture imaging is that gaze imaging can be realized without scanning, and the imaging time is basically equal to the integration time. Through the optimal design of working frequency, bandwidth, integration time, number and arrangement of antennas, the contradiction between detection sensitivity, resolution and imaging time can be balanced.

**Figure 1:**
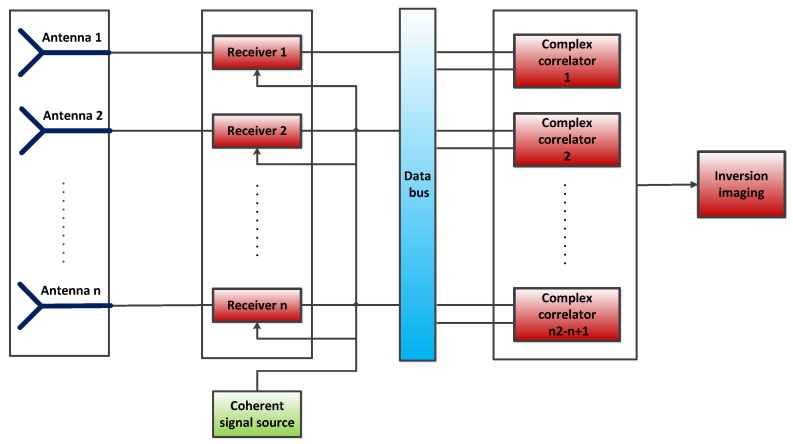
GSAMR system composition block diagram.

The basic unit of a synthetic aperture microwave radiometer is a binary interferometer. Binary interferometer is mainly used to measure the phase difference of the signal of the same target reaching two receivers at different positions. The Synthetic Aperture Microwave Radiometer recovers an estimate of the temperature distribution in the observation area by inverting the interferometric results (complex correlation processing) of different baselines. The synthetic aperture microwave radiometer system consists of an array antenna, array coherent receivers, a combined complex correlator array, signal control, and inverse imaging. Herein, we designed a ground-based synthetic aperture microwave radiometer (GSAMR) detection and imaging system for aircraft takeoff and landing. Its block diagram is shown in Figure 1. To carry out real-time two-dimensional imaging of aircraft, a two-dimensional circular sparse antenna array is recommended. The proposed design consists of a sparse array that is presented below; the sparse array can improve the imaging quality on the premise of determining the number of antenna units. A corrugated horn antenna with high gain and low sidelobe is adopted. Because the system needs a very short integration time, Dick switch or reference source is not used, and full power receiver is selected. Table 1 shows the main parameters of the GSAMR system. The actual requirements of the system in airport applications and its complexity are mainly considered in the demonstration of the index parameters.

Such imaging systems need to operate in real-time with sufficient spatial resolution, detection distance, and sensitivity. The initial goal of the system design is to detect a target with a cross-sectional area of 5 square meters at a distance of 5 km, and the imaging spatial resolution at 1 km can reach 5 m, that is, the radian value of the spatial resolution needs to reach 0.005 rad.

The formula for spatial resolution is:

(1) $$\Delta \theta =\frac{\lambda }{2*D_{\max}}\;\mathrm{rad}$$

where, λ is the working wavelength (0.02 m), and $D_{\max}$ is the farthest distance between the system antenna units. Therefore, the array antenna is arranged on a circular support with a diameter of 2 m, and $D_{\max}$ is equal to 2 m. After a simple calculation, it can be known that the spatial resolution is approximately equal to 0.005 rad.

System sensitivity is calculated as the smallest change at the input that can be detected. The expression for the sensitivity of the aperture synthesizer to observe the extended source is given as

(2) $$\Delta T=\frac{T_{\mathrm{sys}}}{\sqrt{B\tau }}\cdot \frac{A_{\mathrm{syn}}}{n\cdot A_{e}}=\frac{T_{\mathrm{sys}}}{\sqrt{B\tau }}\cdot \frac{D^{2}{}_{\mathrm{syn}}}{n\cdot D^{2}{}_{e}}$$

where, $T_{\mathrm{sys}}$ is the system noise, which is estimated to be 380 K in this system. B is the bandwidth, and the value is 300 MHz. t is the integration time, which is 10 ms. n is the number of antenna elements, which is 16 in this system. $D_{\mathrm{syn}}$ is the aperture area corresponding to the longest baseline of 2 m. $D_{e}$ is the aperture of a single antenna unit, which is 0.2 m. The system sensitivity was calculated to be 1.37 K. For the application scenario of detecting aerial objects, this sensitivity is sufficient, and the sensitivity value can even be designed to be larger, but a larger sensitivity value will affect the effective detection distance.

At the determined spatial resolution and sensitivity, the detection distance can be obtained,

(3) $$R\leq \frac{1}{\sin(\frac{\theta_{A}}{2})\sqrt{\pi }}\times \sqrt{\frac{A_{T}\cdot \Delta T_{T}}{\Delta T_{\min}}}$$

where, R is the detection distance. $A_{T}$ is the area of the assumed target, and $\Delta T_{T}$ is the brightness temperature difference between the target and the background. Because the effective cross-sectional area of the aircraft is small at a distance, it is estimated at 5 square meters. The brightness temperature of the aircraft itself is estimated at 250 K (in fact, the brightness temperature of the aircraft flying at different altitudes is different, which is related to the atmospheric temperature at the altitude of the aircraft). After a simple calculation, it can be obtained that the detection distance is less than 6800 m, but it meets the design requirements of more than 5 km. The following content mainly introduces the arrangement of the antenna array.

**Figure 2:**
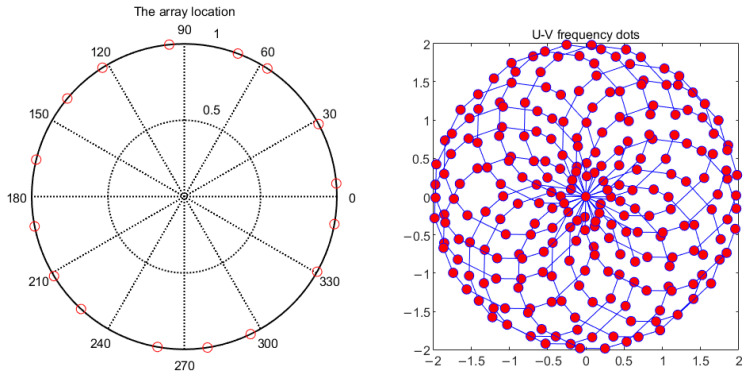
The arrangement of the antenna units and the U-V distribution diagram.

The disadvantage of synthetic aperture is that when using redundant array, the noise of the system increases due to the reduction of array. At the same time, synthetic aperture technology is based on the principle of interferometry, so the antenna beam is usually narrow. Therefore, when designing the array, we should balance the number and redundancy of the array, so as to find a balance between the detection sensitivity and noise temperature of the system. A non-redundant antenna array was used in the GSAMR. For a given number of antennas, the non-redundant antenna array increases the number of spatial frequency sampling points (U-V frequency coverage) and improves the quality of image reconstruction. The specific position of the antenna in the array can be optimized by genetic algorithm. The goal of optimization is to make the independent spatial frequency sampling points as many as possible and evenly distributed [15]. For this application, a two-dimensional circular asymmetric array is preferred. In this array, the antennas are arranged asymmetrically on a circle, and frequency covers as many independent samples as possible. The subsequent results and analyses in this paper are based on two-dimensional circular asymmetric arrays. Table 2 shows the 16-element 2D circular asymmetric array optimized by the GA algorithm. Figure 2 shows the array locations and u-V frequency points.

### 2.2. Inversion Imaging Algorithms

**Figure 3:**
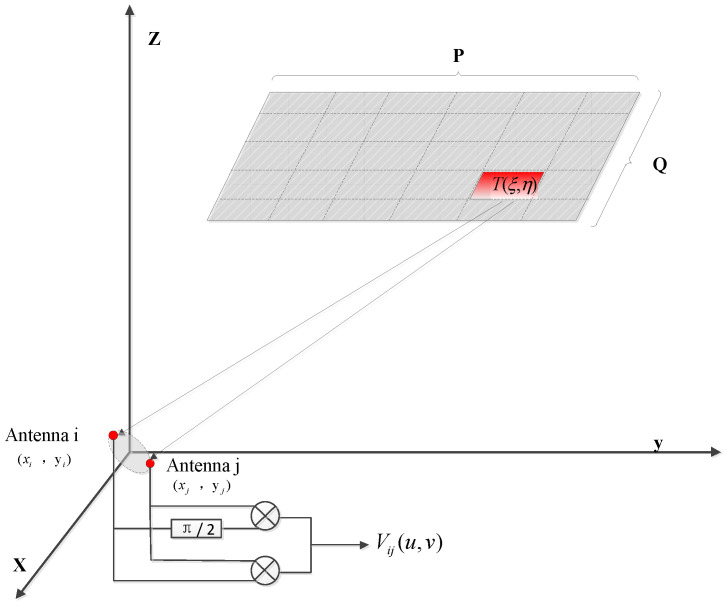
The schematic diagram of binary interferometer measurement.

According to the e relationship between the visibility function and target brightness temperature [16], previous researchers proposed various inversion algorithms, which have been successfully used in sparse antenna array structure imaging systems [17,18]. Stogryn applied the Backus–Gilbert (BG) theory for the inversion of a space-borne mechanical scanning imaging system in 1978 and obtained relatively ideal inversion data [19,20]. The core idea of the BG algorithm is to estimate the inversion brightness temperature by measuring the point source impact response of each baseline. The schematic diagram of binary interferometer measurements is shown in Figure 3.

In an ideal system, the visibility function of the n-element array and target radiation brightness temperature have the following relationship:

(4) $$V_{ij}(u,v)=\frac{1}{\sqrt{\Omega_{i}\Omega_{j}}}\iint_{\xi^{2}+\eta^{2}\leq 1}T(\xi ,\eta )e^{-j2\pi (u\xi +v\eta )}d\xi d\eta$$

where $V_{ij}(u,v)$ is the visibility function corresponding to the *i*-th and *j*-th antennas, which are $n^{2}-n+1$ in total. Its corresponding normalized sampling point coordinates are (*u_ij_*,*v_ij_*), and $\left(x_{i},y_{i}\right)$ and $\left(x_{j},y_{j}\right)$ are the coordinates of the *i*-th and *j*-th antenna elements, respectively. T(ξ,η) is the true brightness temperature at point (ξ,η) in the detection region, and $u_{ij}=(x_{i}-x_{j})/\lambda$, $v_{ij}=(y_{i}-y_{j})/\lambda$. $\Omega_{i,j}$ is the antenna’s solid angle.

In matrix form, Equation (4) can be expressed as

(5) V=G·T

where, $G_{i,j}$ is $\frac{1}{\sqrt{\Omega_{i}\Omega_{j}}}e^{-j2\pi (u\xi +v\eta )}$.

According to the Backus–Gilbert method, the retrieved brightness temperature can be written as

(6) $$\hat{T}=C\cdot V=G^{T}(GG^{T})\cdot V$$

where $C=G^{T}(GG^{T})$ is the pseudo-inverse of *G*-matrix. In this method, the *G*-matrix is regarded as non-ideal, and the real *G*-matrix is obtained using the impact response of the point source in the laboratory. When the *G*-matrix is measured and used in software, the inversion brightness temperature can be obtained through Equation (6).

### 2.3. Error Compensation Method Based on Visibility Function

The actual detection system will have channel, antenna, position, pattern, and temperature errors, etc., [15]. The common method is to correct the *G*-matrix through standard point sources in the laboratory [21,22,23]. However, in airport applications, moving the system to the laboratory regularly to correct the *G*-matrix is impractical. Therefore, the *G*-matrix is considered to be a fixed value in this study (it is usually necessary to conduct laboratory calibration before use), and the error of the visibility function can be corrected by regularly observing the sun in practice to ensure the long-term effectiveness of the system. A simpler and more convenient calibration method is used in this study. We first calibrate the system in the laboratory by a known signal transmitting source from a fixed position. During long-term observation at the airport, the gain and phase of the system will drift, which will reduce the effect of previous calibration. Therefore, we will use non cooperative sources (such as the sun) for secondary error calibration. GeoSTAR’s solar transit observations are made at JPL, which simply point the antenna skyward and let the sun pass through the FOV (field of view) for a few hours. Using solar transit observations, the GeoSTAR antenna pattern errors are obtained. However, using the sun as a reference source to calibrate the magnitude and phase errors of the entire system is not its main job.

In the calibration process, first track the preset sun position parameters in the software, aim at the sun, and then observe for a period of time to obtain new error calibration parameters. The core idea is that, in the final analysis, the system error can be expressed as the amplitude error and phase error of the visibility function. Hence, as long as the output amplitude and phase errors of the visibility function are calculated, all errors can be collectively compensated for. In this method, error calibration is performed before inversion imaging; thus, it is suitable for any subsequent inversion algorithm. Assuming that the detection area is divided into *P* × *Q* grids, *T* is the two-dimensional matrix of row *P* and column *Q*, and the expression of the actual inversion brightness temperature can be written in the discretized form

(7) $$\hat{T}(\xi ,\eta )=\frac{1}{P\times Q}\sum_{i=1}^{n}\sum_{j=i}^{n}V_{ij}(u,v)e^{j\Delta \theta }$$

where,

(8) $$\Delta \theta =2\pi (u_{ij}\xi +v_{ij}\eta )$$

Further, Equation (7) can be written as,

(9) $$\hat{T}(\xi ,\eta )=\frac{1}{P\times Q}\sum_{i=1}^{n}\sum_{j=i}^{n}\left(V_{ij}{}^{I}(u,v)\cdot \cos(\Delta \theta )+V_{ij}{}^{Q}(u,v)\cdot \sin(\Delta \theta )\right)$$

where, $V_{ij}{}^{I}$ and $V_{ij}{}^{Q}$ are two orthogonal signals of the visibility function V, $V_{ij}(u,v)=V_{ij}{}^{I}(u,v)+jV_{ij}{}^{Q}(u,v)$. According to Equation (4), the following relationship can be deduced,

(10) $$\left\{ \begin{array}{l} V_{ij}{}^{I}(u,v)=\sum_{l=1}^{P}\sum_{m=1}^{Q}T(\xi ,\eta )\cdot \cos(\Delta \theta )\\ V_{ij}{}^{Q}(u,v)=\sum_{l=1}^{P}\sum_{m=1}^{Q}T(\xi ,\eta )\cdot \sin(\Delta \theta ) \end{array}\right.$$

Equation (10) applies to the ideal system. In practical systems, it usually contains signal amplitude error and phase error. When the amplitude error and phase error are included, the I path and Q path of the visibility function can be represented by $V_{ij}{}^{{I\;}^{\prime }}(u,v)$ and $V_{ij}{}^{{Q\;}^{\prime }}(u,v)$, respectively, and the above formula can be transformed into

(11) $$\left\{ \begin{array}{l} V_{ij}{}^{{I\;}^{\prime }}(u,v)=A_{ij}\sum_{l=1}^{P}\sum_{m=1}^{Q}T(\xi ,\eta )\cdot \cos(\Delta \theta +\delta_{ij})\\ V_{ij}{}^{{Q\;}^{\prime }}(u,v)=A_{ij}\sum_{l=1}^{P}\sum_{m=1}^{Q}T(\xi ,\eta )\cdot \sin(\Delta \theta +\delta_{ij}) \end{array}\right.$$

where $A_{ij}$ is the amplitude error and $\delta_{ij}$ is the phase error. The function on the right side of the equation can be expanded as

(12) $$\left\{ \begin{array}{l} V_{ij}{}^{{I\;}^{\prime }}(u,v)=A_{ij}\sum_{l=1}^{P}\sum_{m=1}^{Q}T(\xi ,\eta )\cdot \cos(\Delta \theta +\delta_{ij})\\ =A_{ij}\sum_{l=1}^{P}\sum_{m=1}^{Q}T(\xi ,\eta )\cdot \cos(\Delta \theta )\cos(\delta_{ij})-A_{ij}\sum_{l=1}^{P}\sum_{m=1}^{Q}T(\xi ,\eta )\cdot \sin(\Delta \theta )\sin(\delta_{ij})\\ V_{ij}{}^{{Q\;}^{\prime }}(u,v)=A_{ij}\sum_{l=1}^{P}\sum_{m=1}^{Q}T(\xi ,\eta )\cdot \sin(\Delta \theta +\delta_{ij})\\ =A_{ij}\sum_{l=1}^{P}\sum_{m=1}^{Q}T(\xi ,\eta )\cdot \sin(\Delta \theta )\cos(\delta_{ij})+A_{ij}\sum_{l=1}^{P}\sum_{m=1}^{Q}T(\xi ,\eta )\cdot \cos(\Delta \theta )\sin(\delta_{ij}) \end{array}\right.$$

The amplitude error $A_{ij}$ can be obtained by normalizing the maximum output value of the visibility function of each path, which is easy to compensate. Therefore, this study only compensates for the phase error.

From observations, it is known that $\sum_{l=1}^{P}\sum_{m=1}^{Q}T(\xi ,\eta )\cdot \cos(\Delta \theta )$ and $\sum_{l=1}^{P}\sum_{m=1}^{Q}T(\xi ,\eta )\cdot \sin(\Delta \theta )$ in the above formula are the real and imaginary parts of the visibility function without error, respectively; hence, it can be concluded that

(13) $$\left\{ \begin{array}{l} V_{ij}{}^{{I\;}^{\prime }}(u,v)=A_{ij}\cdot (V_{ij}{}^{I}(u,v)\cos\delta_{ij}-V_{ij}{}^{Q}(u,v)\sin\delta_{ij})\\ V_{ij}{}^{{Q\;}^{\prime }}(u,v)=A_{ij}\cdot (V_{ij}{}^{I}(u,v)\sin\delta_{ij}+V_{ij}{}^{Q}(u,v)\cos\delta_{ij}) \end{array}\right.$$

In the formula, $V_{ij}{}^{{I\;}^{\prime }}(u,v)$ and $V_{ij}{}^{{Q\;}^{\prime }}(u,v)$ are measured moderate functions with errors, while $A_{ij}$ and $\delta_{ij}$ are measured errors. Thus, the visibility function after error removal is

(14) $$\left\{ \begin{array}{l} V_{ij}{}^{I}(u,v)=\frac{V_{ij}{}^{{Q\;}^{\prime }}(u,v)\sin\delta_{ij}+V_{ij}{}^{{I\;}^{\prime }}(u,v)\cos\delta_{ij}}{A_{ij}}\\ V_{ij}{}^{Q}(u,v)=\frac{V_{ij}{}^{{Q\;}^{\prime }}(u,v)\cos\delta_{ij}-V_{ij}{}^{{I\;}^{\prime }}(u,v)\sin\delta_{ij}}{A_{ij}} \end{array}\right.$$

**Figure 4:**
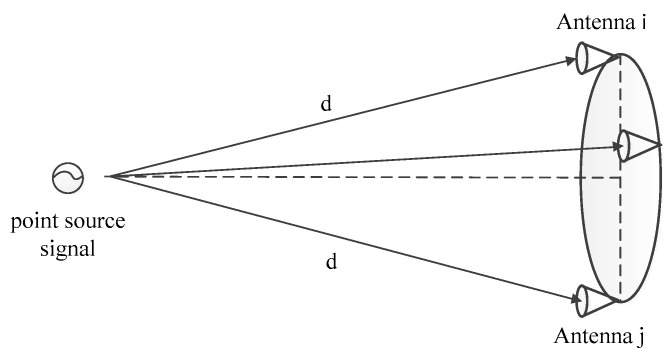
Phase correction diagram.

From the above, Equation (14) can be used for error compensation when $V_{ij}{}^{{I\;}^{\prime }}(u,v)$, $V_{ij}{}^{{Q\;}^{\prime }}(u,v)$, and $\delta_{ij}$ are measured. $V_{ij}{}^{{I\;}^{\prime }}(u,v)$ and $V_{ij}{}^{{Q\;}^{\prime }}(u,v)$ can be obtained directly from the output of the correlator. The main problem to solve is the calculation of $\delta_{ij}$. Since it is very difficult to measure the error between various channels and there are many errors that are difficult to distinguish, a reference point source can be used to calculate $\delta_{ij}$ through the difference between the measured visibility function of the reference point source and the ideal case, as shown in the schematic diagram of Figure 4.

Natural radiative sources such as the sun can be used as the reference point source. When the center of the circular array is aligned with the reference point source, the distance D from each antenna array element to the reference point source is the same because the antenna array element is located on the circumference. Therefore, the Δθ of the visibility function corresponding to the baselines formed by each pair of antennas should be zero. However, the output Δθ of the actual system will not be zero as it will have some phase error $\delta_{ij}$. $\delta_{ij}$ can be obtained from the measured visibility function using numerical calculations to compensate the visibility function.

Assuming that the voltage values of I and Q of the visibility function measured by the reference source are $V_{I}$ and $V_{Q}$, respectively, when the antenna center is aligned with the reference source, the phase error $\delta_{ij}$ is

(15) $$\tan\delta_{ij}=\frac{V_{I}}{V_{Q}}$$

**Figure 5:**
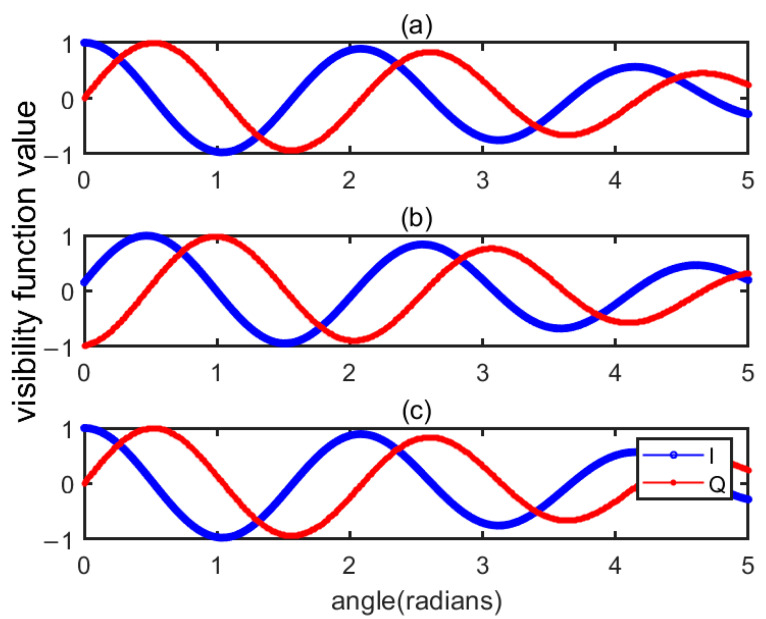
Comparison of visibility function before and after compensating error. (**a**) is the visibility function without the phase error, (**b**) is the visibility function assuming a 30-degree phase error, and (**c**) is the visibility function after compensating for the phase error.

Figure 5 shows the visibility function with errors and the results after compensation. The abscissa of the figure is the angle at which the antenna beam deviates from the center of the calibration source. Figure 5a–c are the visibility function without the phase error, assuming a 30-degree phase error, and after compensating for the phase error using Equation (15). It can be seen from the figure that the visibility function after compensation is consistent with that under the ideal condition, and the compensation method is correct and effective. 

## 3. Results and Discussion

### 3.1. Simulation Verification

**Figure 6:**
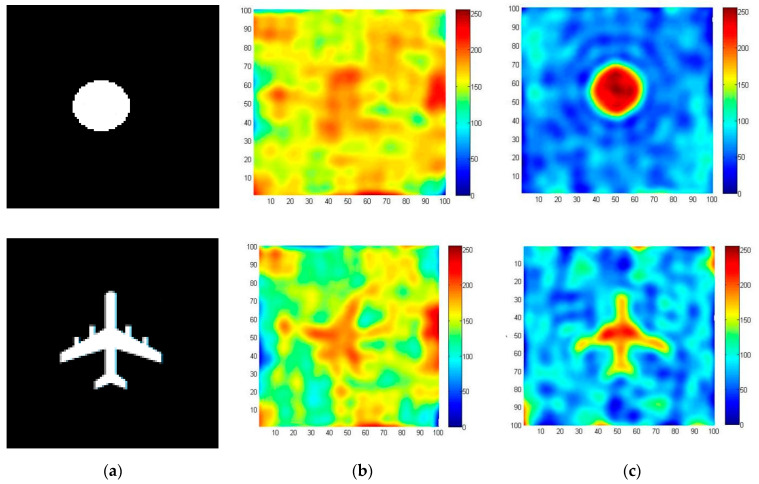
Simulation experiments of different objects. (**a**) Shows the simulated objects, (**b**) shows the results of the system error, and (**c**) is the result after error correction.

**Figure 7:**
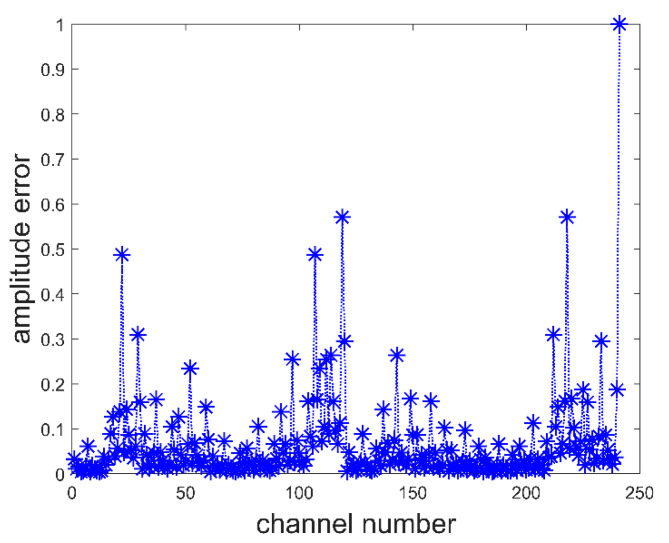
The channel amplitude imbalance error.

**Figure 8:**
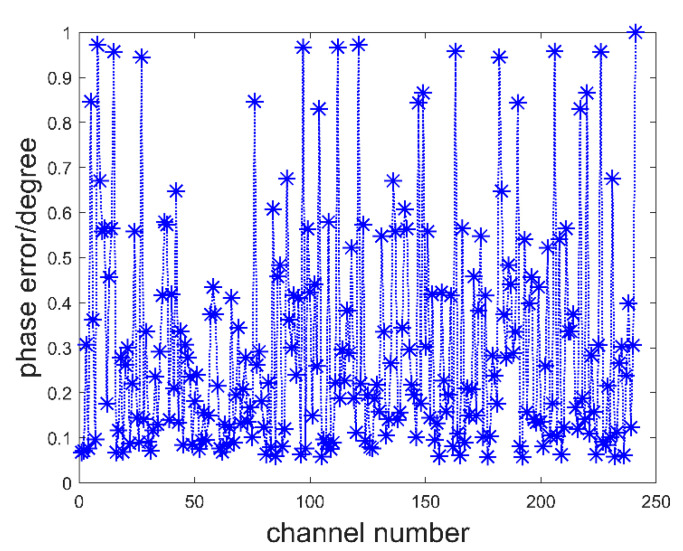
The channel phase error.

To verify the correctness of the system and error correction algorithm, a target imaging simulation experiment was carried out. In the simulation, the targets were assumed to be discs and aircraft. To simplify the simulation, we assumed that the target is an object with a fixed equivalent brightness temperature of 250 K. The background brightness temperature is assumed to be 2.7 K, which is caused by cosmic noise. Figure 6a shows the simulated objects. The effects of regular and irregular objects can be verified with discs and flying machines, respectively. In the simulation process, it was assumed that the system errors were the channel amplitude imbalance error between 0 and 1 times and channel phase error between 0 and 1 degree. The simulated error values for each channel are shown in Figure 7 and Figure 8.

Figure 6 and Figure 7 show the imaging results. The image is 100 pixels × 100 pixels. Figure 6b shows the results of the system error. It is almost impossible to determine the position and shape of the object in the figure. Figure 6c is the result of applying the error correction algorithm in Section 3. As can be seen from the figure, the imaging position of the object is correct and the contour of the image is clear. Therefore, the error correction algorithm in Section 3 can eliminate the amplitude and phase errors of the system, resulting in better results.

### 3.2. Experimental Result

**Figure 9:**
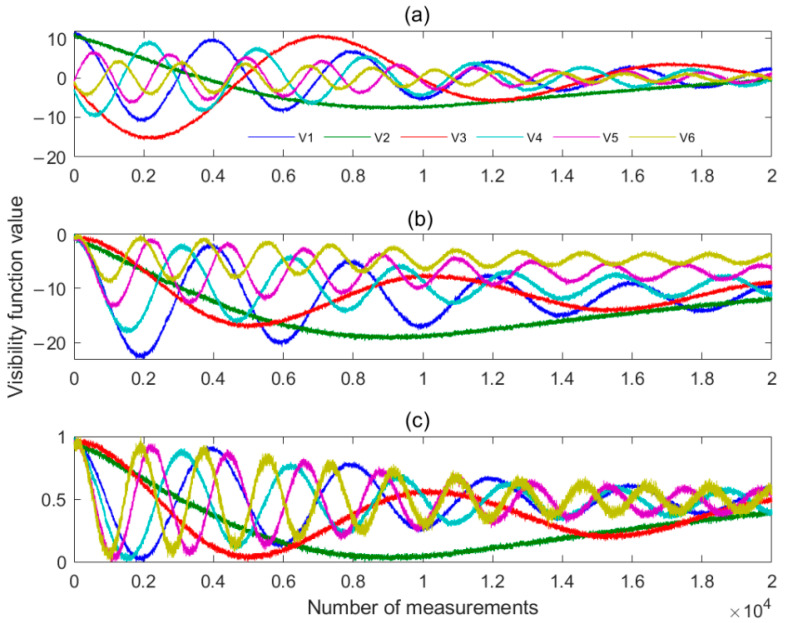
Visibility function calibration process. (**a**) is the raw visibility function, (**b**) is the visibility function after calibration for phase error, and (**c**) is the visibility function after calibration for magnitude error.

After the simulation experiment, we conducted a civil aircraft detection experiment near Qingdao Liuting Airport. Due to experimental conditions, the system’s antenna was not perfectly aligned with the vehicle’s flight path. But the results are still exciting. To monitor fast-moving aircraft, we set the sampling period to 10 ms. First, we observed the sun for a short time, and the collected raw visibility function is shown in Figure 9a. Figure 9b is the visibility function after calibration for phase error using the method in Section 3, and Figure 9c is the visibility function after calibration for magnitude error. To make the display less cluttered, only the visibility functions for the first 6 channels are drawn(V1~V6). As can be seen from the figure, the visibility function error of the system has been calibrated and can be used for aircraft detection.

**Figure 10:**
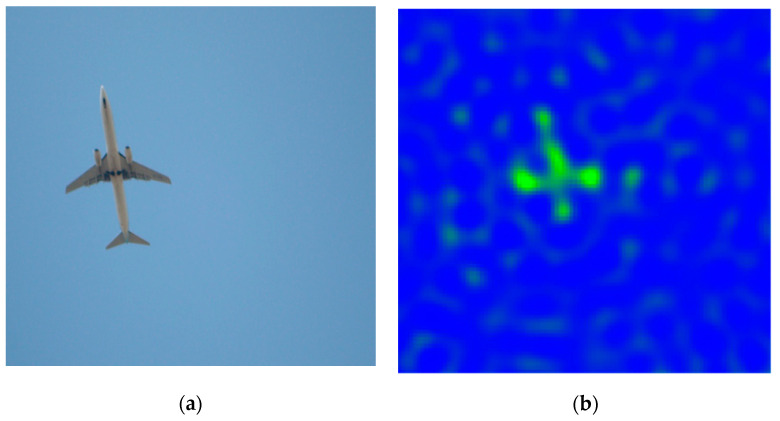
Optical imaging and GSAMR imaging of aircraft. (**a**) Is the image of the aircraft taken using the optical device, (**b**) is the image result of the simultaneous GSAMR system.

We captured the results of the plane’s landing. At that time, the flight altitude of the aircraft was about 1 km, and the aircraft stayed in the detection area for about 2 s. Therefore, the speed of the aircraft at that time is expected to be no more than 60 m/s (about 120 m in the detection area corresponding to the aircraft altitude at that time). Figure 10a is the image of the aircraft taken using the optical device as the aircraft passes, and Figure 10b is the image result of the simultaneous GSAMR system.

It can be seen from the figure that the imaging system is consistent with the overall shape of the aircraft. This can facilitate the ground air traffic control personnel to judge the flight attitude of the aircraft and give suggestions for auxiliary landing. The imaging of the wings on both sides shows that the signal is relatively strong. This is because the radiation brightness temperature of the engine in the wing is higher than that of the other parts of the aircraft. In special weather, especially cloudy and foggy days, the GSAMR aircraft monitoring system is very useful because, in these scenarios, optical methods and human eyes cannot detect the aircraft in this scenario, and the GSAMR system can monitor the altitude of the aircraft during take-off and landing in real-time every 10 ms.

Through the experimental results of the system, the initial intention of the project team was realized, that is to design and verify the feasibility of the synthetic aperture passive detection system in the aircraft-assisted landing. As mentioned above, synthetic aperture microwave radiation technology has been widely used in spaceborne ground detection and observation of static objects such as buildings on the ground. This technology is rarely used for the detection of fast moving flying targets, which is one of the novelties of this paper, and provides readers with a new research idea. It should be emphasized that the author believes that this passive detection method is an effective supplement to the active detection method, rather than a substitute for active detection technology and equipment. Active radar has clear advantages in most cases, especially when the stealth and cost of the detection system are not a concern. After continuous research and improvement, synthetic aperture microwave radiation technology has great application prospects in the future. In addition to the assisted take-off and landing of civil aircraft, it also has great development potential in stealth target detection.

At present, most of the systems adopting the passive detection technology of synthetic aperture microwave radiometers adopt the arrangement of redundant arrays, which can reduce the noise of the system. The design of this system is different, considering that the background of the aircraft is the sky, and the background noise (the theoretical minimum value of 2.7 K on a sunny day) is much lower than the radiation intensity of the aircraft. Therefore, in the design of this system, a non-redundant array is selected, and under a fixed number of receiving antennas, the most spatial frequency sampling can be obtained. The system array arrangement given in this paper is not necessarily the best, it can only be said that in the application of aircraft detection, the detection effect and construction cost are balanced. In the follow-up research, the number of antennas will be further increased, and the advantages and disadvantages of different antenna arrangements will be studied in depth.

## 4. Conclusions

This study introduces a GSAMR system for real-time monitoring of civil aviation aircraft during take-off and landing and proposes a simple and fast error correction method suitable for airport application scenarios. Finally, the simulation and experimental results are given, and the real-time image of the aircraft is obtained in the experiment. The altitude of the aircraft obtained using the proposed imaging method is consistent with that obtained by optical photography, which proves that this technique can be used for real-time monitoring of civil aviation aircraft during take-off and landing and is a practical method for assisting landing. The advantage of this technique is that it can detect normally on cloudy days and is suitable for all-weather observation. The GSAMR system meets the expected requirements of our project, but there remains room for improvement. Further studies focused on miniaturizing the system and improving the performance of the inversion algorithm are required to make the system practical and popular for future airport-assisted landing. In addition, when the aircraft and sun exist simultaneously in a field of view, the sun as the background will inevitably affect the detection effect of the aircraft. We have not yet quantitatively assessed this impact. However, we believe that in a short time, compared with high-speed aircraft (moving targets), the sun can be considered stationary, so we can eliminate the background influence of the sun by signal processing or image processing. In the follow-up research work, we will focus on this work.

## Figures and Tables

**Table 1:** Main Specifications of GSAMR.

Parameter	Specifications
Operating Frequency	15.2 GHz
antenna elements	16
bandwidth	300 MHz
integration time	10 ms
Brightness temperature measurement range	5–350 K
System sensitivity	≤2 K (at 10 ms)
Spatial resolution	0.005 rad
Detection distance	≥5 km

**Table 2:** 16 cells two-dimensional circle-asymmetric array.

Antennas	Location (Radian)			
16	0.086	1.670	3.338	4.865
	0.495	2.137	3.689	5.162
	0.995	2.444	3.969	5.769
	1.212	2.897	4.536	6.102

## References

[1] Le Vine D., Swift C., Haken M. (2001). Development of the synthetic aperture microwave radiometer, ESTAR. IEEE Trans. Geosci. Remote Sens..

[2] Kerr Y., Waldteufel P., Wigneron J., Delwart S., Cabot F., Boutin J., Escorihuela M., Font J., Reul N., Gruhier C. (2010). The SMOS Mission: New Tool for Monitoring Key Elements of the Global Water Cycle. Proc. IEEE.

[3] Brown M., Torres F., Corbella I., Colliander A. (2008). SMOS Calibration. IEEE Trans. Geosci. Remote Sens..

[4] Lemmetyinen J., Uusitalo J., Kainulainen J., Rautiainen K., Fabritius N., Levander M., Kangas V., Greus H., Pihlflyckt J., Kontu A. (2007). SMOS Calibration Subsystem. IEEE Trans. Geosci. Remote Sens..

[5] Tanner A., Wilson W., Lambrigsten B., Dinardo S., Brown S., Kangaslahti P., Gaier T., Ruf C., Gross S., Lim B. (2007). Initial Results of the Geostationary Synthetic Thinned Array Radiometer (GeoSTAR) Demonstrator Instrument. IEEE Trans. Geosci. Remote Sens..

[6] Torres F., Tanner A., Brown S., Lambrigsten B. (2007). Analysis of Array Distortion in a Microwave Interferometric Radiometer: Application to the GeoSTAR Project. IEEE Trans. Geosci. Remote Sens..

[7] Zhang C., Liu H., Yan J.Y., Sun W.Y., Zhang S.W., Liu H.G., Wu J. (2010). Imaging Algorithm and Experimental Demonstration of Rotating Scanning Interferometric Radiometer. Proceedings of the 2010 IEEE International Geoscience and Remote Sensing Symposium.

[8] Chen K., Zhu Y., Guo X., Guo W., Li Q., Gui L., Ni W. (2010). Design of 8 mm-band Aperture Synthetic Radiometer and Imaging Experiment. J. Infrared Millim. Terahertz Waves.

[9] Chen L., Li Q., Yi G., Zhu Y. (2013). One-Dimensional Mirrored Interferometric Aperture Synthesis: Performances, Simulation, and Experiments. IEEE Trans. Geosci. Remote Sens..

[10] Dong J., Li Q., Jin R., Zhu Y., Huang Q., Gui L. (2010). A Method for Seeking Low-Redundancy Large Linear Arrays in Aperture Synthesis Microwave Radiometers. IEEE Trans. Antennas Propag..

[11] Yujiri L., Shoucri M., Moffa P. (2003). Passive millimeter-wave imaging. IEEE Microw. Mag..

[12] Lettington A. (2005). Design and development of a high-performance passive millimeter-wave imager for aeronautical applications. Opt. Eng..

[13] Le Vine D.M. (1990). The sensitivity of synthetic aperture radiometers for remote sensing applications from space. Radio Sci..

[14] Ruf C.S., Swift C.T., Tanner A.B., Le Vine D.M. (1988). Interferometric synthetic aperture microwave radiometry for remote sensing of the earth. IEEE Trans. Geosci. Remote Sens..

[15] Chen H., Chao K., Zhao Z., Lang R., Jin Y. Analysis of two-dimensional circle-array synthetic aperture microwave radiometer channel errors. Proceedings of the 2010 International Conference on Microwave and Millimeter Wave Technology.

[16] Corbella I., Torres F., Camps A., Duffo N., Vall-llossera M., Rautiainen K., Martin-Neira M., Colliander A. (2005). Analysis of correlation and total power radiometer front-ends using noise waves. IEEE Trans. Geosci. Remote Sens..

[17] Li H., Liu H., Wu J., Niu L., Zhang C. (2013). Research on the Preprocessing Method for the Visibility Functions in Synthetic Aperture Radiometer and the Time Domain Simulation. J. Electron. Inf. Technol..

[18] Sun F., Zhang S. (2014). Fast Image Reconstruction for Non-uniform Sampling of Thinned Array of Synthesis Aperture Radiometer. J. Electron. Inf. Technol..

[19] Stogryn A. (1978). Estimates of brightness temperatures from scanning radiometer data. IEEE Trans. Antennas Propag..

[20] Liu H., Wu J., Wu Q. Some further consideration for the image retrieving of synthetic aperture radiometer. Proceedings of the 24th Asian Conference on Remote Sensing (ACRS).

[21] Ruf C. (1991). Error analysis of image reconstruction by a synthetic aperture interferometric radiometer. Radio Sci..

[22] Chen J., Li Y., Wang J., Li Y., Zhang Y. (2014). Regularization Imaging Algorithm with Accurate G Matrix for Near-Field Mmw Synthetic Aperture Imaging Radiometer. Prog. Electromagn. Res. B.

[23] Yao X. (2013). Analysis and Correction the Inter-channel Mismatch of Synthetic Aperture Radiometer. TELKOMNIKA Indones. J. Electr. Eng..

